# Aquaporin mRNA in Human Saliva

**DOI:** 10.3390/genes16070804

**Published:** 2025-07-08

**Authors:** Katharina Rump, Daria Pakosch-Nowak, Andrea Witowski, Bjoern Koos, Dominik Ziehe, Jennifer Orlowski, Michael Adamzik, Martin Kunkel, Markus Baumann

**Affiliations:** 1 Klinik für Anästhesiologie Intensivmedizin und Schmertherapie, Knappschaft Kliniken Universitätsklinikum Bochum, 44892 Bochum, Germany; andrea.witowski@knappschaft-kliniken.de (A.W.); bjoern.koos@rub.de (B.K.); dominik.ziehe@rub.de (D.Z.); jennifer.orlowski@rub.de (J.O.); michael.adamzik@knappschaft-kliniken.de (M.A.); 2 Klinik für Mund-, Kiefer- und Plastische Gesichtschirurgie, Knappschaft Kliniken Universitätsklinikum Bochum, 44892 Bochum, Germany; daria.pakosch-nowak@knappschaft-kliniken.de (D.P.-N.); martin.kunkel@knappschaft-kliniken.de (M.K.); 3 Zahnarztpraxis Sprockhövel, 45549 Sprockhövel, Germany; praxis@dr-markus-baumann.de

**Keywords:** AQP5, AQP1, AQP9, saliva, oral samples, RNA levels

## Abstract

Background: Aquaporins (AQPs) are integral membrane proteins that facilitate water transport across biological membranes. While their role is well-characterized in various tissues, their function in the oral cavity remains poorly understood. Saliva is an easily accessible, non-invasive biofluid that contains stable extracellular RNA and can reflect both systemic and local physiological or pathological processes, making it a promising source for RNA analyses. This study investigates AQP mRNA levels in human saliva. Methods: Saliva samples were collected from patients of a dental practice and analyzed using quantitative PCR to detect AQP levels. An *in silico* analysis of AQPs in cells of the oral cavity were performed. Baseline data of the patients were recorded. Results: Our findings demonstrate the presence of multiple AQP subtypes in human saliva. AQP5 was the most abundant, followed by AQP9 and AQP1. The levels of several AQPs showed intercorrelation, whereas AQP3 appeared to be independently regulated and did not correlate with the other AQPs. Conclusions: This study demonstrates that differential AQP mRNA levels can be detected in human saliva. These findings suggest that salivary AQP mRNA may serve as surrogate markers for altered AQP levels in cells of the oral cavity. In the future, such patterns of AQP levels could potentially be used to identify or monitor pathological conditions affecting the oral mucosa or salivary glands. Further studies are required to validate this approach and to understand its diagnostic relevance.

## 1. Introduction

Aquaporins (AQPs) are a family of integral membrane proteins that function as water channels, facilitating the rapid and selective transport of water and, in some cases, small molecules such as glycerol and urea across cell membranes [1]. These proteins play a crucial role in a variety of physiological processes, including the regulation of water balance in various tissues and organs and the regulation of cell migration [2]. In saliva and salivary glands, aquaporins are particularly important as they influence the secretion and composition of saliva, which is essential for oral hygiene, digestion, and the protection of the oral mucosa [3].

Saliva is a complex biological fluid consisting of more than 99% water and less than 1% proteins, electrolytes, and other low-molecular-weight components [4]. It is primarily produced by the major salivary glands (parotid, submandibular, and sublingual), with additional contributions from numerous minor glands distributed throughout the oral cavity. Gingival crevicular fluid contributes only minimally to total salivary composition [4].

Owing to its easy, non-invasive accessibility and constant contact with teeth and periodontal tissues, saliva offers an attractive medium for biomarker discovery. Numerous studies have identified salivary biomarkers linked not only to oral but also to systemic diseases [5]. Molecules such as DNA, RNA, proteins, and metabolites—typically detected in blood—are also present in saliva, supporting its use in early disease detection and therapeutic monitoring [4].

Saliva and buccal samples are increasingly employed in biomedical research, including studies utilizing modern *omics* technologies. In addition to soluble molecules, these samples contain a considerable number of cells, predominantly a mixture of buccal epithelial cells and leukocytes [6]. This cellular composition is expected, given the buccal mucosa’s high permeability and rich vascular supply [6].

Furthermore, thousands of human mRNAs have been identified in cell-free saliva. Microarray and quantitative PCR (qPCR) analyses have enabled the creation of a reference database of salivary mRNA profiles from healthy individuals [7]. These findings underpin the emerging concept of Salivary Transcriptome Diagnostics (STD) as a powerful and non-invasive tool for both disease diagnostics and ongoing health surveillance [7].

The RNA of aquaporins detected in saliva may originate from various cellular sources, including salivary gland cell turnover, buccal epithelial cell shedding, shed cells from the tongue, immune cells, or extracellular vesicles such as exosomes [8]. To date, systematic studies investigating the presence and mRNA patterns of aquaporins in human saliva are lacking.

A detailed study of AQP mRNA in saliva could help us understand how saliva is produced and how it relates to diseases like Sjögren’s syndrome, dry mouth, and dental problems such as cavities and gum disease. It may serve as a non-invasive tool to monitor gene expression in salivary glands or nearby tissues, or to detect problems with oral health. Protein levels in saliva, however, can be influenced by many factors after the genes are expressed [3,9,10]. In this paper, we present a comprehensive and systematic investigation of AQP mRNA in whole saliva samples. This work could potentially help identify AQP markers based on altered AQP mRNA levels in saliva, which may indicate the presence of diseases in the oral cavity that are associated with changes in AQP expression.

## 2. Materials and Methods

### 2.1. Study Design and Cohort

The OKAPI study (German Clinical Trial Registry No. DRKS00032425) prospectively enrolled patients who met the inclusion criteria. The study received approval from the Ethics Committee of the Medical Faculty of the Ruhr University Bochum (23-7821-BR, approval date: 7 June 2023) and the Ethics Committee of the Westphalia-Lippe Medical Association (2023-416-b-S, approval date: 26 July 2023). The study protocol, site-specific informed consent forms, participant education and recruitment materials, and other required documents, including any subsequent modifications, were reviewed and approved by these ethical review bodies. The study was conducted in accordance with the revised Declaration of Helsinki, good clinical practice guidelines, and local regulatory requirements. Patients were recruited over a period of 17 months at a dental practice after providing written informed consent. Eligible participants were adult dental patients aged 18 to 75 years. Exclusion criteria included genetically determined structural disorders of the dental hard tissue (e.g., amelogenesis imperfecta, dentinogenesis imperfecta, odontogenesis imperfecta), dementia and/or psychotic illness, lack of capacity to consent, and insufficient knowledge of the German language to understand the study’s scope and participation requirements.

### 2.2. Baseline Characteristics

Baseline characteristics were collected by a questionnaire. This survey includes the following measurement variables:

- Age;
- Gender (m/f/d);
- Pre-existing conditions: arterial hypertension, [non-]insulin-dependent diabetes mellitus;
- Cardiovascular diseases [including CHD, post-myocardial infarction, PAD, etc.];
- Nicotine abuse;
- Current or previous malignant tumor disease: localization, TNM stage, radiotherapy.

Patients were recruited in a dental practice, resulting in a cohort of 204 patients. Most of the patients (59.8%) were females and the median age was 46.5 years (Table 1).

### 2.3. Collection of Samples

As part of the dental treatment at the dental practice, 2 mL saliva samples were obtained from patients included in the study. The saliva collection was performed throughout the whole day. Patients were asked to rinse their mouth with water and wait for 10 min before collection. The saliva samples were collected using Saliva RNA Collection and Preservation Devices (Norgen Biotek, Thorold, ON, Canada disturbed by BioCAT, Heidelberg, Germany according to the manufacturer’s instructions. RNA was then isolated from the saliva samples using the Total RNA Purification Kit (Norgen Biotek, Thorold, ON, Canada, disturbed by BioCAT, Heidelberg, Germany). Following isolation, the samples were stored at −80 °C.

### 2.4. RNA Quantification

For the cDNA synthesis, 1 µg of RNA was utilized with the High-Capacity cDNA Reverse Transcription Kit (Thermo Fisher Scientific, Wilmington, NC, USA) to translate RNA into cDNA. The cDNA was then analyzed using qPCR with specific primers (MWG eurofins, Ebersberg, Germany) for each AQP (Table 2). The levels were quantified using the ΔCt method, with β-Actin (ACTB) serving as the reference gene, as described previously [11,12].

### 2.5. In Silico Analysis

Since the study material included whole saliva samples, we performed an *in silico* analysis to investigate the expression patterns of various aquaporins in cell types commonly found in the oral cavity. Our focus was on salivary gland cells, oral squamous epithelium, tongue cells, and immune cells. For this analysis, we used data from the Human Protein Atlas, specifically the normalized transcripts per million (nTPM) values from the Consensus dataset. To analyze the tissue-specific expression of various aquaporins (AQPs), data from the Human Protein Atlas (HPA, https://www.proteinatlas.org/, Version 24.0, accessed on 15 May 2025) was utilized [13,14]. First, each individual AQP (e.g., AQP1, AQP2, etc.) was searched using the HPA search function. In the “Tissue RNA expression” section, the “RNA Expression Overview” was selected. The “Consensus dataset” and the “Organ Expression Alphabetical” view were used to obtain an alphabetical overview of organ and tissue expression. For each tissue of interest (e.g., salivary glands), the corresponding bar charts were examined, and the normalized transcripts per million (nTPM) values were extracted and recorded.

### 2.6. Statistical Analysis

Patient characteristics are reported as percentages for categorical variables and as means with standard deviations (SDs) or medians with interquartile ranges (25th and 75th percentiles), as appropriate. Categorical variables were compared using McNemar or Fisher’s exact tests. Continuous independent variables were compared using Student’s *t*-test or the Mann–Whitney U test, following normal distribution assessment with the Shapiro–Wilk test. A *p*-value of less than 0.05 was considered significant. Unless otherwise stated, data are presented as mean ± standard deviation (SD) values. All analyses were performed using SPSS (version 28, IBM, Chicago, IL, USA). Graphical presentations were created using GraphPad Prism 9 (GraphPad, San Diego, CA, USA).

## 3. Results

**Figure 1:**
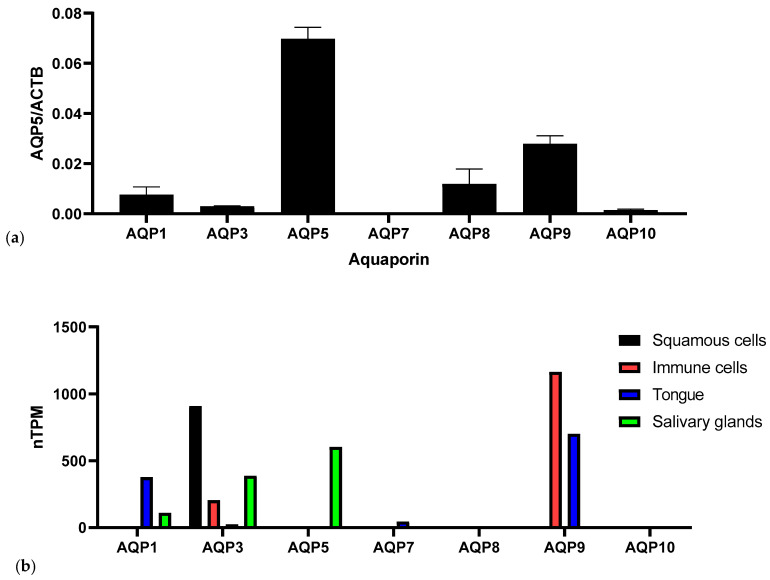
(**a**) Levels of different AQPs in saliva samples of patients of a dental practice (*n* = 204). The levels of the different AQPs were measured by qPCR and normalized to beta-Actin (ACTB); (**b**) normalized transcripts per million (nTPM) of different AQPs in squamous cells, immune cells, tongue, and salivary glands according to human protein atlas.

In the initial step, the mRNA levels of various aquaporins in saliva were analyzed. Among these, AQP5 exhibited the highest levels, with levels more than double those of AQP9, the second most abundant aquaporin. AQP8 and AQP1 followed AQP9 in abundance levels. The lowest levels observed in our study were for AQP3, AQP10, and AQP7 (Figure 1a), and AQP4 could not be detected at all. As we used whole saliva samples for our analysis, the cellular distribution of AQPs in oral cavity cells was analyzed using *in silico* analysis with data from the “Human Protein Atlas” (Figure 1b). In squamous cells only AQP3 is expressed, which can also be found in immune cells (T-cells), similarly to AQP9 (neutrophils). In cells of the tongue mainly AQP1 and AQP9 can be found. The most abundant AQP in salivary glands is AQP5, followed by AQP3 and AQP8 (Figure 1b).

**Figure 2:**
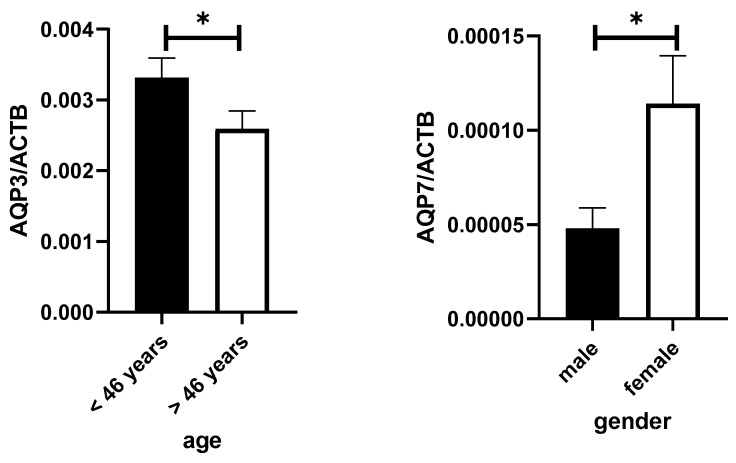
AQP3 mRNA levels stratified by median age of the cohort and AQP7 mRNA level in saliva stratified by gender. The levels of the different AQPs were measured by qPCR and normalized to beta-Actin (ACTB). *: *p* < 0.05.

In the second step, this study examined whether the levels of these aquaporins were influenced by age, gender, or nicotine use. For most aquaporins, no significant associations were found. However, the level of AQP7 showed a strong dependence on gender (*p* = 0.0125; see Figure 2), and the level of AQP3 decreased with age (*p* = 0.038; Figure 2).

**Figure 3:**
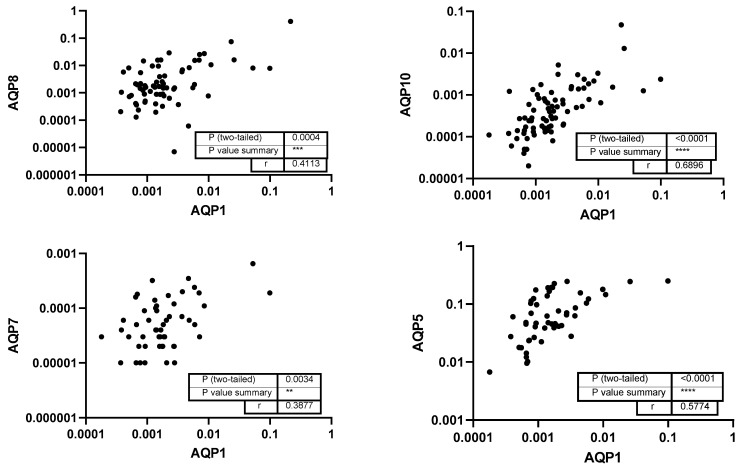
Correlation of AQP1 with the levels of other aquaporins. Each data point represents a paired expression value from the mRNA levels of two different aquaporins (AQPs). The expression level of AQP1 measured by qPCR (normalized to ACTB) is plotted at the *x*-axis and the levels of the other AQPs can be found at the *y*-axis. **: *p* < 0.01; ***: *p* < 0.001; ****: *p* < 0.0001.

**Figure 4:**
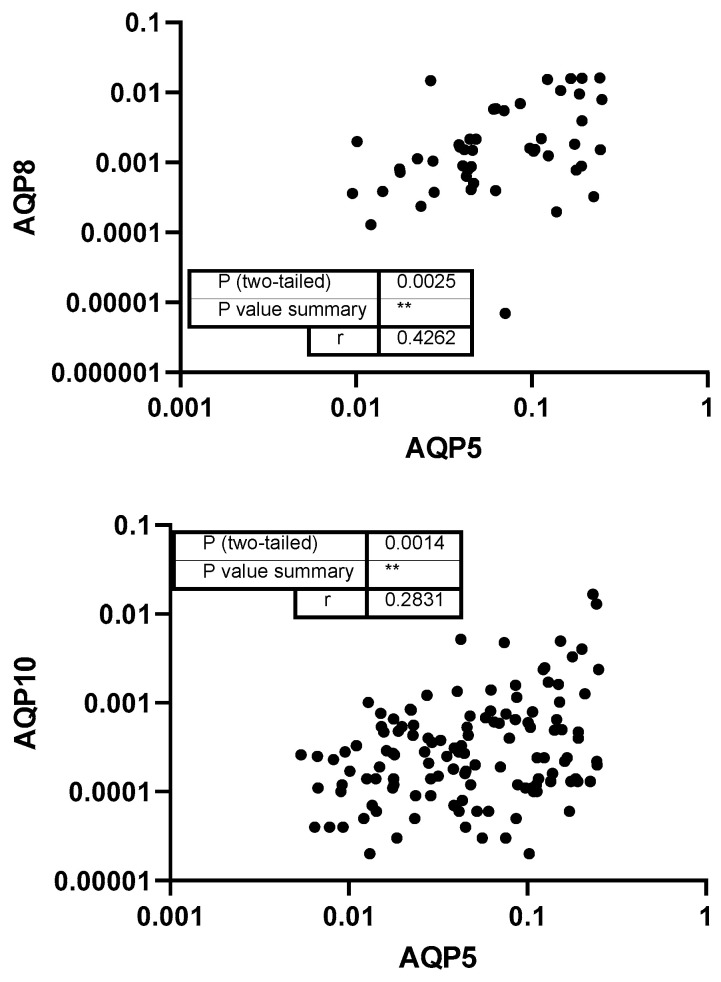
Correlation of AQP5 to AQP8 and AQP10. Each data point represents a paired expression value from the mRNA levels of two different aq-uaporins (AQPs). The expression level of AQP5 measured by qPCR (normalized to ACTB) is plotted at the *x*-axis and the levels of the other AQPs can be found at the *y*-axis. **: *p* < 0.01.

Subsequently, the study investigated the correlations between the levels of different aquaporins. Analyzing correlations between different aquaporins can reveal shared regulatory mechanisms, functional interactions, or compensatory roles in water and solute transport within the oral cavity. A strong correlation was found between the levels of AQP1 and AQP10 (*r* = 0.6896) and AQP5 (*r* = 0.5774), whereas AQP1 and AQP8 and AQP7 showed moderate correlations (*p* < 0.001; Figure 3). Additionally, AQP5 showed weak correlation with AQP10 and a moderate correlation with AQP8 (*p* = 0.004; Figure 4). No correlation was detected between the level of AQP3 and any other aquaporins.

**Figure 5:**
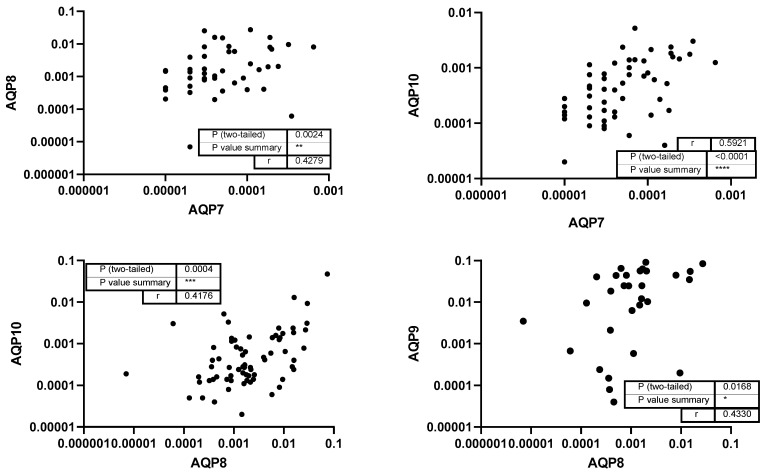
Correlation of AQP7 and AQP8 to AQPs 9 and 10. Each data point represents a paired expression value from the mRNA levels of two different aq-uaporins (AQPs). The expression levels of AQP7 and AQP8 measured by qPCR (normalized to ACTB) are plotted on the *x*-axis, and the levels of the other AQPs can be found on the *y*-axis. *: *p* < 0.05; **: *p* < 0.01; ***: *p* < 0.001; ****: *p* < 0.0001.

Furthermore, moderate correlations between AQP7 to AQP8 (*p* = 0.0024) and AQP10 (*p* < 0.0001) as well as AQP8 to AQP9 (*p* = 0.0168) and AQP10 (*p* = 0.0004) could be detected (Figure 5).

A correlation matrix was created to visually represent the correlations (Table 2). Again, AQP3 appears to be independent of other aquaporins, whereas AQP1 and AQP10 show the strongest correlations (Table 3).

## 4. Discussion

In this study, we systematically examined the levels of AQPs in saliva. The most frequently abundant aquaporin was found to be AQP5. To explore potential origins of aquaporins in saliva, we performed an *in silico* analysis of cells within the oral cavity. Our data suggest that AQP5 may primarily originate from salivary gland cells, AQP9 from immune cells and the tongue, AQP1 from the tongue, and AQP3 from squamous epithelial cells.

AQP5 is commonly found in glandular tissues such as the salivary glands, lacrimal glands, and pancreas [15]. In the salivary gland, AQP5 is located at the apical membrane, including the intercellular secretory canaliculi of acinar cells [16]. Thus, the AQP5 mRNA level detected in this study might mainly originate from salivary gland cells. Hence, mRNA in saliva does not have a functional role locally, but reflects transcriptional activity of source cells (epithelial, glandular, immune). In this context, the aquaporins have mainly been examined in salivary glands [17]. The expression of aquaporin mRNA in salivary glands is a complex and finely tuned process regulated by various endogenous and exogenous factors [18]. Aquaporin-5 (AQP5), in particular, is known for its central role in saliva production [18]. Alterations in the expression and function of AQP5 and other aquaporins can lead to a range of pathological conditions, including Sjögren’s syndrome, an autoimmune disease characterized by dry eyes and dry mouth [19,20].

The second most frequently expressed aquaporin in saliva samples was AQP9. As AQP9 is mainly expressed in immune cells (neutrophils) or in cells of the tongue, the detected mRNA could have its origin in these cells [6,21]. To date, little is known about AQP9 in saliva. However, there are indications that patients with Sjögren’s syndrome (SS) have autoantibodies against AQP9 [19]. In addition, autoantibodies targeting other aquaporins have recently been identified. In a study of 34 SS patients, 13 individuals (38.2%) had autoantibodies directed against extracellular domains of various AQPs [19]. Specifically, autoantibodies were detected against AQP1 (*n* = 2), AQP3 (*n* = 1), AQP8 (*n* = 6), and AQP9 (*n* = 4), whereas no antibodies were found against AQP4 or AQP5. Each patient had antibodies to only one extracellular domain. AQP binding was further verified by radioimmunoassay with intact AQPs, Western blots, and AQP-transfected cells. Patients with anti-AQP antibodies had more severe xerophthalmia compared with anti-AQP-negative patients, suggesting a potential pathogenic role of these antibodies [19].

The presence of AQP8 in saliva is not surprising. In digestive glands, AQP8 is primarily expressed in the parotid and salivary glands, as well as the liver and pancreas. In rat parotid, submandibular, and sublingual glands, AQP8 is found in myoepithelial cells surrounding the acini and intercalated ducts, rather than in the acinar or ductal cells [22]. Hence the presence of AQP8 mRNA in saliva might originate from exfoliated epithelial and myoepithelial cells of the salivary glands, reflecting either passive shedding or active secretion via extracellular vesicles [23].

AQP8 was followed by AQP1, which has long been recognized for its role in saliva and salivary glands [24]. Earlier studies demonstrated that adenovirus encoding human aquaporin-1 (AdhAQP1) administration to rat submandibular glands resulted in a two- to threefold increase in salivary secretion compared to glands treated with a control virus. These findings suggest that hAQP1 gene transfer could serve as a promising approach for the treatment of post-radiation salivary hypofunction by increasing AQP1 expression in the salivary glands [25]. In addition, AQP1 seems to be expressed in cells of the tongue as indicated by our *in silico* data.

AQP3, which can be found in immune cells (T-cells), squamous cells, and also in cells of the salivary glands [26], has also been previously detected in saliva samples [9]. AQP3 mRNA is likely important for saliva secretion, because it is consistently expressed at significant levels in all major human salivary glands and localized to the basolateral membranes of both mucous and serous acinar cells [27]. Together with AQP5, which is found on the luminal membrane, AQP3 is thought to contribute to transcellular osmotic water flow, facilitating the movement of water from the interstitium through acinar cells into the forming primary saliva. Therefore, AQP3 may play a crucial supportive role in the generation of saliva by enabling water entry into secretory cells [27]. Another study confirmed that salivary glands express and secrete AQP3 proteins into whole saliva. Moreover, participants with xerostomia had higher salivary levels of AQP3 than those without xerostomia. According to Ichiyama et al. [10], the mRNA expression of AQP3 is crucial for saliva secretion. Compared to healthy controls, patients with Sjögren’s syndrome and dry mouth had lower mRNA expression but higher immunoreactive intensities of AQP3 in their salivary glands [10]. In their study, AQP3 concentration was higher in xerostomic participants compared to non-xerostomic participants, which could be due to the evaluation of secreted protein levels in saliva rather than mRNA expression in salivary gland tissue samples, as performed in another study [10]. The secreted protein level may not correspond with mRNA expression due to post-translational and post-transcriptional pathways [28,29].

In our study, the levels of AQP7 and AQP10 was quite low. Research on the function of AQP10 and AQP12 in the digestive system, particularly in the oral cavity, remains limited [16,22].

Another notable observation was that AQP7 levels were higher in women than in men. The sex-specific expression of AQP7 has been demonstrated in other studies. In adipose tissue, only females showed an increased abundance of AQP7 [30]. This could be caused by estrogen binding to the AQP7 promoter. Estrogen induces AQP7 expression by binding to estrogen response elements (EREs) in the promoter of the Aqp7 gene, resulting in fat catabolism in adipocytes [31]. Furthermore, gonadal steroids play a crucial role in determining sex-dependent fat distribution and accumulation, and studies have shown that they can influence AQP7 expression. Specifically, estrogen response elements in the promoter region of the AQP7 gene, which lead to fat catabolism in adipocytes, may provide an explanation for the onset of obesity during menopause [32].

In our study, only AQP3 seemed to be downregulated by age, which is in line with another study, where no significant differences were found when dividing the AQP mRNA expression data by age [33].

One of the most important findings of our study was that the levels of the different AQPs are correlated. Another study found that in healthy tissues, various aquaporins (AQPs) correlated with each other, with AQP3, AQP5, and AQP9 showing significant associations. However, in tumor tissues, most correlations were lost, with AQP1 showing no correlation with other AQPs, indicating that carcinogenesis disrupts the coordinated regulation of AQP gene expression in healthy tissues [33]. In particular, the strong correlation of AQP3 found in the aforementioned study was in contrast to our results, where AQP3 seemed to be independent of other AQPs. Several studies have demonstrated that the expression of different AQPs can be co-regulated by factors such as hormonal regulation, osmotic stress, and inflammatory signals [34]. Hormones like vasopressin and aldosterone play a crucial role in modulating AQP expression in tissues such as the kidney, while osmotic changes drive a coordinated regulation of multiple AQPs to maintain cellular and systemic water balance [34]. Furthermore, inflammatory cytokines have been shown to influence the expression of various AQPs, particularly in organs like the lung and gastrointestinal tract [35,36]. The observation of correlated AQP mRNA levels in whole saliva—despite its mixed cellular origin—suggests that salivary RNA reflects coordinated glandular gene expression programs and may serve as a surrogate for studying regulatory changes in salivary pathophysiology

The correlation in AQP expression levels can arise due to co-regulation or because AQPs are involved in similar physiological processes. Regarding aspects related to the co-regulation of multiple AQPs, possibly reflecting compensatory mechanisms in pathological versus physiological conditions, a previous study demonstrated that AQP7, AQP8, and AQP9 are co-regulated in different regions of the canine epididymis, with marked upregulation in the caput and downregulation in the cauda under cryptorchid conditions. This coordinated expression pattern suggests a compensatory role of these AQPs in response to pathophysiological alterations in the luminal microenvironment [37]. In addition, tissue-specific expression patterns often lead to coordinated expression of multiple AQPs, as seen in the kidney, where AQPs such as AQP1, AQP2, and AQP3 are expressed in different nephron segments to regulate water reabsorption [34]. Similarly, in pathological conditions such as cancer, the upregulation or downregulation of multiple AQPs can occur simultaneously, highlighting their potential roles in tumor growth and metastasis [38].

Research findings further support this notion. In the kidney, AQPs like AQP1, AQP2, AQP3, and AQP4 have been shown to be co-regulated by factors such as vasopressin and osmotic stress [39,40,41]. In the lung, AQPs including AQP1, AQP3, and AQP5 display correlated expression patterns in response to inflammatory signals, suggesting their involvement in pulmonary fluid regulation [42]. Moreover, in various cancers, AQPs such as AQP1, AQP3, and AQP5 exhibit correlated expression, reinforcing their significance in tumor progression.

Some limitations of our study should be acknowledged. First, this study only uses whole saliva from the oral cavity of dental patients. Hence, we cannot pinpoint where the mRNA we measure is initially expressed. A detailed analysis of AQP mRNA levels in saliva may provide indirect insights into salivary gland gene regulation and could serve as a non-invasive biomarker for changes in epithelial homeostasis or glandular dysfunction. In addition, the timing of our sample collection may not have been optimal, as samples were obtained throughout the whole day, following only the recommendations provided in the manufacturer’s protocol. However, as saliva composition can fluctuate over the course of the day and in response to food or fluid intake, the timing and pre-analytical conditions of sample collection may affect RNA content and should have been carefully controlled or documented in salivary biomarker studies [43,44,45].

In conclusion, the levels of different AQPs can be both co-regulated and correlated depending on the physiological or pathological context. While co-regulation often occurs through common regulatory mechanisms, correlation in expression levels reflects their involvement in shared biological processes. Future research should further elucidate the regulatory networks governing AQP expression, which could provide deeper insights into their roles in health and disease. A potential intervention to regulate AQPs is the use of microRNAs (miRNAs). miRNAs can specifically target AQP mRNAs, leading to their degradation or translational repression, thereby modulating AQP expression levels [46]. This approach offers a promising strategy for controlling AQP activity in various physiological and pathological conditions, including salivary gland dysfunction, cancer, and water balance disorders.

## 5. Conclusions

This study provides novel insights into the levels of aquaporin mRNA (AQPs) in human saliva. Our findings demonstrate that multiple AQP subtypes are present in saliva, with AQP5 being the most abundant form. These findings contribute to a better understanding of salivary gland function and may have clinical implications for disorders such as xerostomia and salivary gland dysfunction. Future research should explore the potential of AQPs as therapeutic targets to improve saliva secretion in patients with impaired salivary gland function.

## Figures and Tables

**Table 1:** Baseline characteristics of the study cohort.

	*n* = 204
Gender male	82 (40.2%)
Age median (IQR)	46.5 (35–56.75)
Arterial hypertonia (yes)	30 (14.75)
Heart disease	11 (5.4%)
Smoking (yes)	46 (22.5%)
Smoking in years	4.85 ± 11.3
Diabetes	5 (2.5%)
Cancer	3 (1.5%)

**Table 2:** Primers utilized for qPCR.

Primer Name	Sequence
AQP1_Rt_Se	GCCATCCTCTCAGGCATCAC
AQP1_RT_Human_AS	GTAGCCAGCACGCATAGCAC
AQP3_Rt2_Se	GGAATAGTTTTTGGGCTGTA
AQP3_Rt2_AS	GGCTGTGCCTATGAACTGGT
AQP5_RT_2_SE	TCGGTTCAGCCCCGCTCACT
AQP5_RT_2_AS	GCCACACGCTCACTCAGGCT
AQP7_Rt2_Se	GGACTGGGGACACAGGGATA
AQP7_Rt2_As	GCTGAAAGTGCAATCCACGG
AQP8_RT_SE	GAGATCATCCTGACGACGCT
AQP8_RT_AS	TTCATGCAGCCTCCAGACAC
AQP9_RT_SE_neu	GCGAACGCATTTGCAGATCA
AQP9_RT_AS_neu	CAACCAAAGGGCCCACTACA
AQP10_RT_SE	CTACGTGGGTGGTAACGTCTC
AQP10_RT_AS	TAGGTGGCAAAAATGGAGGCT

**Table 3:** Correlation matrix. Dark blue squares indicate strong positive correlations, whereas red squares indicate strong negative correlations. The corresponding correlation values (r) associated with each color can be found in the ‘color scheme’ column.

	AQP8	AQP10	AQP1	AQP5	AQP7	AQP9	AQP3	Color Scheme
AQP8								0.7
AQP10								0.6
AQP1								0.45
AQP5								0.3
AQP7								0.15
AQP9								0
AQP3								−0.1

## Data Availability

The data presented in this study are available on request from the corresponding author.
